# Paste-Level Evaluation of a Hybrid Silicomanganese Slag–Steel Slag–OPC-Activated Binder: Mechanical Performance, Simplified Carbon Footprint and Mn Leaching Reduction

**DOI:** 10.3390/ma19091891

**Published:** 2026-05-04

**Authors:** Junku Duan, Xuanshuo Zhang, Jing Zhao, Shudong Hua, Hongbo Li

**Affiliations:** 1 Ningxia Huasheng Energy Saving and Environmental Protection Technology Co., Ltd., Yinchuan 750021, China; szswhy@163.com (J.D.); ytmn0420@gmail.com (S.H.); 2 College of Civil and Hydraulic Engineering, Ningxia University, Yinchuan 750021, China; z17341244772@163.com (J.Z.); 13469578416@nxu.edu.cn (H.L.)

**Keywords:** silicomanganese slag, alkali-activation, mechanical property, ion exchange, carbon intensity

## Abstract

Silicomanganese slag (SiMnS), a Mn-bearing by-product from silicomanganese alloy production, is often stockpiled in large quantities and may pose environmental concerns due to potential metal leaching. This study develops an OPC-rich hybrid SiMnS–steel slag–fly ash–OPC-activated composite binder, referred to as SMSAB, in which OPC accounts for 55% of the solid precursor mass. Different alkali contents and sodium silicate moduli were investigated, and the optimised paste was characterised in terms of mechanical strength, reaction products, pore structure, carbon-footprint and heavy-metal leaching. The best performance was obtained at an alkali content of 4% and a sodium silicate modulus of 1.0, giving 28-day compressive and flexural strengths of 65.13 MPa and 3.37 MPa, respectively. XRD, SEM-EDS, FTIR and MIP results showed that the main reaction products were C-(A)-S-H, N-A-S-H and C-N-A-S-H gels, which refined the pore structure and produced a dense matrix. The reduction in Mn leaching may be associated with physical encapsulation, possible charge-balancing interactions within gel structures, changes in Mn-related bonding environments and the presence of Mn-bearing phases. Leaching concentrations of Zn, Mn, Cr, Cu and Ni satisfied the Grade III groundwater limits used in China. The calculated carbon intensity of SMSAB was 3.97 kg·(m^3^·MPa)^−1^, indicating a favourable strength-to-emission balance compared with the reference systems considered. It should be noted that the present work examines paste specimens only; aggregate skeleton, traffic loading, freeze–thaw cycling and wet–dry/moisture cycling were not included. Therefore, the results demonstrate binder-level potential rather than direct qualification of SMSAB as a pavement base or subbase material.

## 1. Introduction

Ordinary Portland Cement (OPC) is the primary construction binder in modern society, and its production entails the extraction and consumption of a substantial amount of raw materials and energy, resulting in a significant carbon footprint [1]. Specifically, global OPC production accounts for approximately 2.3 billion tonnes of carbon emissions annually (6% to 8% of global emissions), contributing to the greenhouse effect and global warming [2,3,4]. To mitigate the environmental damage caused by cement production, it is necessary to develop eco-friendly alternative binders that promote cleaner production and sustainable development in the construction industry. Fortunately, a green binder known as alkali-activated materials (AAMs) has been developed [5,6,7,8]. Research has indicated that AAMs offer significant economic and environmental benefits by reducing carbon emissions while preserving natural resources required for OPC production [9,10]. Moreover, AAMs possess various characteristics such as high mechanical strength, durability, and excellent erosion control capabilities [11]. Due to these advantages, AAMs are considered promising alternative binder systems to OPC.

Nevertheless, AAMs also face several practical challenges that should not be overlooked. Depending on precursor chemistry, activator type and curing/exposure conditions, AAMs may suffer from efflorescence caused by alkali migration and carbonate formation, relatively high autogenous or drying shrinkage, sensitivity to curing conditions, handling issues associated with alkaline activators, and uncertain long-term durability under some aggressive environments. These limitations are particularly relevant for road engineering, where the binder may be exposed to moisture fluctuation, de-icing salts, freeze–thaw action and repeated traffic loading. Therefore, the development of new AAM systems should consider not only strength and carbon reduction, but also environmental safety, durability and practical constructability.

AAMs are synthesised by combining aluminosilicate precursor materials with chemical activators. Hertel et al. [12] described AAMs as inorganic binder systems characterised by three-dimensional aluminosilicate networks (N-A-S-H type gel, representing alkaline cations). This network forms when the aluminosilicate material is activated in a high-concentration alkaline solution (hydroxide or silicate). Because of this reaction feature, a wide range of precursor materials can be used to produce alkali-activated binders, including industrial by-products rich in silica-, alumina- and calcium-bearing phases [13]. Consequently, AAMs offer potential pathways for transforming diverse industrial by-products into beneficial resources. Over the past decade, numerous studies have investigated the feasibility of using different industrial by-products as precursors for AAMs, with emphasis on mechanical strength, durability and formation mechanisms, including fly ash (FA), blast furnace slag and metakaolin [14,15,16]. Recently, data-driven methods have also been introduced into the design and performance prediction of alkali-activated materials. For example, Kazemi et al. developed an active-learning-assisted stacked machine learning model to predict the compressive strength of alkali-activated ultra-high-performance concrete, showing the potential of machine learning tools for reducing experimental workload and supporting mixture optimisation [17]. However, the availability, chemical reactivity and regional supply of commonly used precursors such as fly ash and blast furnace slag vary considerably. This has encouraged the exploration of other industrial solid wastes as potential binder components, including silicomanganese slag (SiMnS).

SiMnS is an industrial waste slag formed during the smelting of manganese steel cast iron to produce silicon–manganese alloy [18]. Recent data indicate that global SiMnS production reached approximately 23.3 million tonnes in 2020, with SiMnS production in China accounting for about 57.1% of the global total [19]. Large amounts of SiMnS are stockpiled in open areas, which may pose risks to the surrounding ecological environment. Due to its high-temperature quenching process, SiMnS contains aluminosilicate phases and may show potential reactivity under alkaline activation. Therefore, SiMnS also has the potential to be a precursor material for AAMs. However, compared with fly ash- or blast furnace slag-based AAMs, SiMnS-based alkali-activated materials have received relatively limited attention. Kumar et al. [20] found that mechanical activation improved the reaction degree of SiMnS. Nath and Kumar [21] developed alkali-activated binders using FA and SiMnS as co-precursors at room temperature and attributed the strength improvement to enhanced CaO reactivity in SiMnS and increased gel formation. Navarro et al. [22] optimised the sodium-silicate activation conditions of SiMnS and reported acceptable workability. Recent studies have shown that alkali-activated SiMnS development is mainly composed of three steps: the formation of Si-rich gel, the formation of Al-rich gel, and the enhancement of gel polymerisation degree and maturation [19]. Similarly, Guedes et al. [23] demonstrated that the development of SiMnS alkali-activated binders at room conditions using NaOH activators is feasible from the perspective of rheology and micromorphology. Previous studies have demonstrated the feasibility of SiMnS-based AAMs by focusing mainly on mechanical activation, SiMnS–FA blended systems, activator optimisation, early reaction kinetics, rheology and microstructural development. However, several aspects remain insufficiently addressed. First, the use of SiMnS together with high-alkalinity steel slag as a co-precursor has rarely been examined, although steel slag can provide additional Ca-bearing phases that may promote the formation of C-(A)-S-H type gels. Second, the environmental behaviour of Mn-bearing SiMnS in alkali-activated matrices, especially the leaching response and immobilisation mechanism of Mn, has not been systematically discussed. Third, the carbon efficiency of such binders has seldom been evaluated together with mechanical performance. These gaps limit a clear assessment of SiMnS-based binders for engineering use.

Based on our previous work on steel slag-based cementitious materials for pavement applications [24], this study designed a hybrid SiMnS–SS–FA–OPC alkali-activated binder, referred to as SMSAB. The objective was not to directly reproduce a complete pavement base or subbase mixture, but to evaluate the paste-level binder performance and environmental safety before aggregate-level mixture design. The effects of alkali content and sodium silicate modulus on setting time and mechanical strength were first investigated. The reaction products, microstructure and pore characteristics were then analysed using XRD, SEM-EDS, FTIR and MIP. Finally, leaching behaviour and carbon intensity were evaluated to clarify whether the proposed binder can combine mechanical performance, reduced Mn leaching and material-level carbon reduction potential. The results provide a basis for the subsequent development of aggregate-containing road engineering mixtures, where traffic loading, freeze–thaw resistance and wet–dry/moisture cycling should be further verified.

## 2. Materials and Methods

### 2.1. Materials

#### 2.1.1. SiMnS, SS, FA, and OPC

The SiMnS was obtained from the Industrial Solid Waste Resource Utilization Collection Station (Shizuishan, China). The SS was obtained from the converter SS of a steel plant (Yinchuan, China). Since the particle sizes of SiMnS and SS were too large for direct use, both materials were pulverised using a planetary ball mill. In this study, the specific surface areas of SiMnS and SS were 302.6 m^2^/kg and 729.5 m^2^/kg, respectively. The SS alkalinity value was 2.85, classifying it as high alkalinity slag. FA was provided from a thermal power plant (Yinchuan, China). The cement used was 42.5 grade OPC. Sodium silicate (water glass) powder was purchased from Gongyi Borun Refractory Co., Ltd., (Zhengzhou, China) with an initial modulus of 3.15, SiO_2_ content of 9.5 wt.%, Na_2_O content of 29.0 wt.%, and water content of 61.5 wt.%. Sodium hydroxide pellets were purchased from Guangdong Puhui Chemical Raw Materials Reagent Co., Ltd.,(Shaoguan, China) to adjust the water glass modulus, with a purity of 96%.

**Figure 1:**
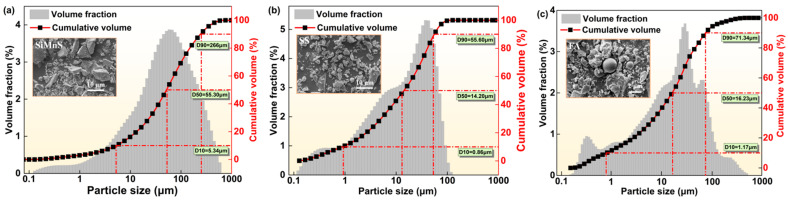
Particle size distribution and micromorphology: (**a**) SiMnS; (**b**) SS; and (**c**) FA.

**Figure 2:**
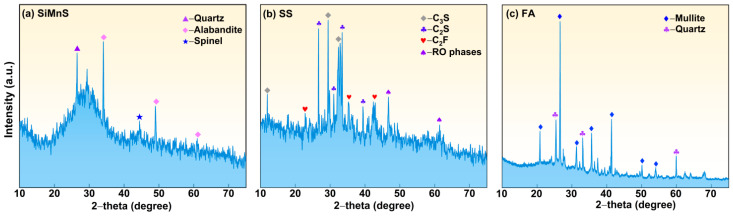
XRD patterns: (**a**) SiMnS; (**b**) SS; and (**c**) FA.

Table 1 shows the chemical composition of the raw materials, as determined using an X-ray fluorescence spectrometer (S2 RANGER LE03040428, Bruker AXS GmbH, Karlsruhe, Germany). The results indicated that SiMnS contained high contents of alumina and silica, suggesting its potential as an alkali-activation precursor. Figure 1 displays the particle size distribution and micromorphology of SiMnS, SS and FA. The microstructure of SiMnS consists of irregular angular particles with a smooth surface. The microstructure of SS contains small particle aggregates (silicic acid modified phase), bright white particles (RO phase) and irregular particles (calcium ferrite phase). Among these, the D50 of SS is the smallest, indicating that SS has finer granularity. Figure 2 shows the XRD patterns of SiMnS, SS and FA. The mineral phases of SiMnS include alabandite (*α*-MnS), spinel (MgAl_2_O_4_) and quartz (SiO_2_). It is worth noting that the presence of α-MnS gives SiMnS its light green colour. In the XRD patterns of SiMnS and SS, broad amorphous humps were observed in the range of 20–35°, indicating the presence of amorphous calcium aluminosilicate phases and their potential reactivity. This amorphous peak was also found in the XRD pattern of FA, representing a characteristic peak of the glassy structures conducive to alkali excitation reaction.

#### 2.1.2. Alkaline Activator Solution

First, sodium hydroxide pellets were dissolved in deionised water and stirred with a magnetic stirrer for about 5 min until they were completely dissolved. Then, the ready-made water glass solution was poured into the sodium hydroxide solution to adjust the modulus and alkali content. Finally, the alkali activator prepared by combining the water glass solution and sodium hydroxide was allowed to stand for 24 h.

### 2.2. Paste Design and Preparation

In this study, the solid precursor composition of SMSAB paste was fixed at SiMnS:SS:FA:OPC = 15:15:15:55 by mass, as shown in Table 2. This composition was selected based on our previous work on steel slag-based cementitious materials for pavement applications [24] and preliminary paste preparation trials, with the aim of maintaining a stable mechanical baseline while incorporating SiMnS, SS and FA as industrial by-products. In the preliminary unactivated SiMnS–SS-based binder system, the paste with W/S = 0.32 reached 28-day compressive and flexural strengths of 50.43 MPa and 2.30 MPa, respectively. This W/S value also allowed the paste to be mixed and cast without visible bleeding or severe loss of compactability. Therefore, W/S = 0.32 was adopted for all mixtures to maintain a constant effective water content and to isolate the effects of activator chemistry.

On this basis, a two-factor, three-level experimental matrix was designed to optimise the alkaline activator. The Na_2_O content was set at 3%, 4% and 5% of the paste mixture mass, and the sodium silicate modulus, defined as the molar ratio of SiO_2_ to Na_2_O, was set at 0.8, 1.0 and 1.2. A total of nine SMSAB paste mixtures were therefore prepared. The optimisation criteria included setting time, 3-day, 7-day and 28-day compressive strength and flexural strength. The optimised mixture was further evaluated in terms of reaction products, pore structure, heavy-metal leaching behaviour and carbon intensity.

**Figure 3:**
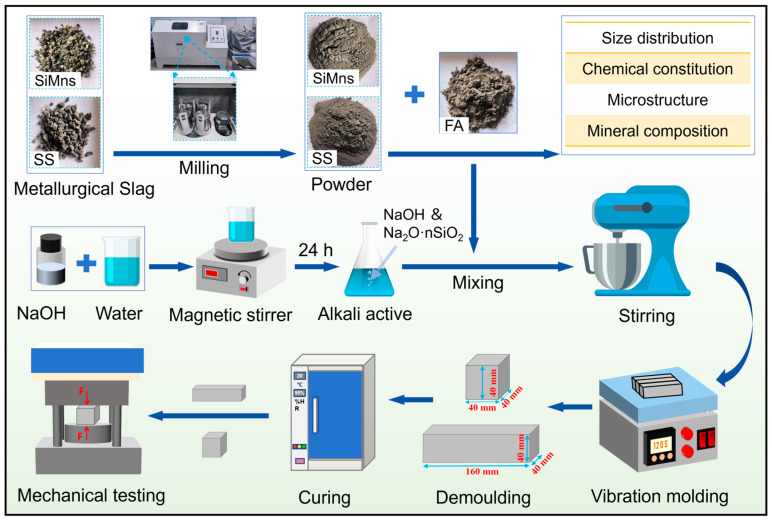
Sample preparation process and detection.

SMSAB paste was prepared according to the mixture proportions described above. The dry raw materials were first mixed uniformly in an automatic mixer. The alkaline activator was then added and mixed with the raw materials to obtain fresh SMSAB paste. After mixing, the fresh paste was cast into 40 mm × 40 mm × 40 mm moulds for compressive-strength testing and 40 mm × 40 mm × 160 mm moulds for flexural-strength testing. All specimens were compacted using a mechanical vibration table. After 24 h of standard curing at 20 ± 2°C and relative humidity above 95%, the specimens were demoulded, sealed in plastic bags, and continuously cured under the same standard conditions until testing. The sample preparation and testing procedure are shown in Figure 3.

### 2.3. Methods

#### 2.3.1. Particle Size Distribution and Specific Surface Area

Because milled SiMnS and SS powders were used in this study, their particle size distribution and specific surface area were measured to support the analysis of the alkali-activation reaction of SMSAB. A planetary ball mill was used for milling SiMnS and SS separately. According to the optimal milling time of the waste slag system reported by Li et al. [25], Sha et al. [26] and Han et al. [27], the milling time of SS was 45 min, and the SiMnS was 70 min. The particle size distribution and specific surface area of the milled SiMnS and SS powder were measured by a Laser Particle Size Analyzer (Mastersizer 3000, Malvern Instruments Ltd., Malvern, Worcestershire, UK) with ethanol as the dispersing medium and detection range of 0.017–2000 μm.

#### 2.3.2. Setting Time

According to the standard for test method of performance on building mortar [28], the setting time of SMSAB paste with normal consistency was detected by the Vicat Apparatus (ZKS-100, Hebei Xin Test Machine Manufacturing Co., Ltd., Cangzhou, China). The instant when the SMSAB mixture was added to water was chosen as the start of the setting time. The instant when the test needle of the Vicat apparatus stopped sinking or sank to the bottom plate at 4 mm ± 1 mm after the initial setting test needle was released for 30 s was chosen as the initial setting time. The instant when the initial setting test needle sank into the specimen surface at 0.5 mm, and the circular cutting edge on the needle did not leave marks on the specimen, was chosen as the final setting time.

#### 2.3.3. Mechanical Properties

According to the test method of cement mortar strength [29], the compressive strength and flexural strength of hardened SMSAB paste samples were tested by a microcomputer-controlled electronic pressure-testing machine (YAW-300D, Jinan Liling Testing Machine Co., Ltd., Jinan, China). The test ages were 3 days, 7 days and 28 days, at least three specimens were tested for each paste, and the loading rate was 2400 N/s.

#### 2.3.4. Microscopic Tests

Several microscopic tests were carried out to further clarify the alkali activation mechanism and strength development behaviour of SMSAB paste. The phase composition of SMSAB paste was investigated by X-ray diffraction (XRD; D8 VENTURE, Bruker AXS SE, Karlsruhe, Germany) with the anode target material being Cu, the scanning range was 3–73°, and the step length was 0.02°. Phase identification was performed by comparing the diffraction peaks with standard powder diffraction files. The microstructure and element distribution of SMSAB paste were investigated by scanning electron microscopy and energy dispersive spectroscopy (SEM-EDS; EVO 18, Carl Zeiss Microscopy GmbH, Jena, Germany) with an acceleration voltage ranging from 0.2 to 30 kV. The infrared absorption characteristics of the SMSAB reaction products were investigated by Fourier transform infrared spectroscopy (FTIR; Avatar 380, Thermo Nicolet Corporation, Madison, WI, USA) with a resolution of 0.5–1 cm^–1^, and scanning was conducted 40 times. The pore structure of SMSAB paste was studied by a mercury intrusion porosimeter (MIP; AutoPore V 9605, Micromeritics Instrument Corporation, Norcross, GA, USA). Powdered samples were used for XRD and FTIR. For SEM observation, fractured surfaces were gold-coated before testing, while small hardened fragments were used for MIP analysis.

#### 2.3.5. Heavy-Metal Element Content and Leaching Tests

Since the paste was composed of SiMnS composites with a high Mn ion content, two standard Chinese leaching tests were conducted on SMSAB and SMSB pastes (after curing for 28 days) to evaluate the environmental risk of heavy-metal leaching following alkali activation. These tests included a solid waste-extraction procedure for leaching toxicity with the horizontal vibration method (HVM) [30] and a solid waste-extraction procedure for leaching toxicity with the sulfuric acid and nitric acid method (SNM) [31].

In the HVM test, deionised water was used as the leaching agent. Both paste samples were oven-dried to constant weight at 105 °C, ground, and sieved through a 3 mm mesh. A 50 g sample was placed in a 2 L wide-mouth bottle, with deionised water added at a solid-to-liquid ratio of 1:10 (V/W). The suspension was mixed on a horizontal oscillation device (frequency 110 ± 10 rpm, amplitude 40 mm) at room temperature for 8 h and allowed to stand for 16 h before filtration of the leachate through a 0.45 μm membrane filter.

In the SNM test, a leaching agent was prepared by diluting a mixture of concentrated sulfuric acid and nitric acid (2:1 mass ratio) to a pH of 3.20 ± 0.05. Both paste samples were also oven-dried to constant weight at 105 °C, ground, and sieved through a 9.5 mm mesh. A 50 g sample was placed in a 2 L wide-mouth bottle, with leaching agent added at a liquid-to-solid ratio of 1:10 (L/kg). The samples were mixed in a rotary shaker at 30 rpm at 23 ± 2 °C for 18 ± 2 h. After rinsing the 0.45 μm membrane filter with 1% dilute nitric acid, the leachate was filtered and collected.

The concentrations of heavy-metal ions in the leachates were determined using inductively coupled plasma mass spectrometry (ICP-MS; Agilent 7900, Agilent Technologies, Inc., Santa Clara, CA, USA). Prior to testing, the leachates were digested with nitric acid. Each sample was measured three times, and the average value was recorded.

## 3. Results and Discussion

### 3.1. Setting Time

**Figure 4:**
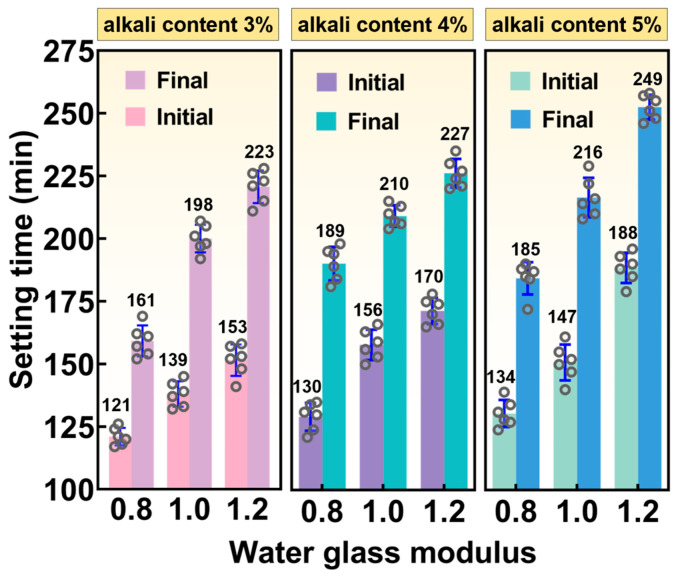
Effect of different water glass modulus and alkali content on setting time.

Figure 4 presents the effects of different water glass modulus and alkali content on the setting time. It can be seen that the setting time of SMSAB paste increased with increasing sodium silicate modulus at the same alkali content. Specifically, when the alkali content was 3%, for SMSAB paste containing 1.0 and 1.2 water glass modulus, the initial setting time increased by 14.88% and 26.45%, and the final setting time increased by 22.98% and 38.51%, respectively. In contrast, under the same water glass, the setting time of SMSAB paste increased slightly with the increase in alkali content. More explicitly, when the water glass was 1.0, the initial setting time of SMSAB slurry with 3% and 4% alkali content increased by 12.23% and 5.76%, respectively, and the final setting time increased by 6.06% and 9.09%, respectively. This indicates that the effect of alkali content is less than that of water glass modulus. In other words, compared with the water glass modulus, alkali content shortened the setting time of SMSAB paste to a certain extent. In fact, the setting time is closely related to the chemical activity of the precursor material. Under alkaline conditions, the amorphous vitreous structure in the precursor dissolves and releases cations such as Ca^2+^, Al^3+^, and Si^4+^, which accelerates the formation of aluminosilicate polymer gels, and thus generates C-(A)-S-H gels with lower solubility, which accelerates the coagulation time of SMSAB paste to a certain extent [32]. This phenomenon aligns with the pattern of influence of a high OH^–^ environment on AAMs paste condensation time reported in the previous literature [33,34].

### 3.2. Mechanical Properties

#### 3.2.1. Compressive Strength

**Figure 5:**
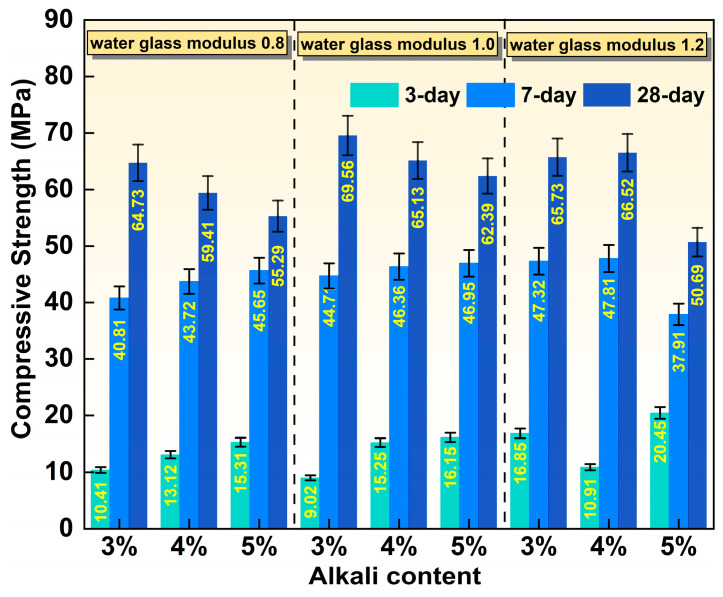
Effect of water glass modulus and alkali content on the compressive strength of SMSAB paste.

Figure 5 illustrates the effect of water glass modulus and alkali content on the compressive strength of SMSAB paste at different ages. It can be observed that when the modulus of water glass was 0.8 and 1.0, the 3-day and 7-day compressive strength of SMSAB paste increased with increasing alkali content. Specifically, when the alkali content increased from 3% to 5%, and the water glass modulus was 0.8, the 3-day compressive strength of SMSAB paste increased by 12.11% and 47.07%, respectively, and the 7-day compressive strength increased by 7.13% and 11.86%, respectively. When the water glass modulus was 1.0, the 3-day compressive strength of SMSAB paste increased by 69.07% and 79.05%, respectively, and the 7-day compressive strength increased by 3.69% and 5.01%, respectively. The above phenomenon may be related to the presence of Ca^2+^ in SS. In the presence of water-glass solution, Ca^2+^ can combine with silica–oxygen tetrahedrons and alumina–oxygen tetrahedrons dissolved in SiMnS and FA to form more hydrated calcium silicate aluminate (C-A-S-H) and hydrated calcium silicate (C-S-H) gels, resulting in an increased strength of the SMSAB paste [24,35]. In addition, the additional nucleation sites for the polymerisation were provided by Ca^2+^, contributing to the generation of amorphous gels, enhancing the early mechanical strength of the SMSAB paste [26]. Differently, when the modulus of water glass was 0.8 and 1.0, the 28-day compressive strength of SMSAB paste decreased with the increasing alkali content. Specifically, when the alkali content increased from 3% to 5%, and the water glass modulus was 0.8, the 28-day compressive strength of SMSAB paste decreased by 8.22% and 14.58%, respectively. When the modulus of water glass was 1.0, the compressive strength was reduced by 6.37% and 10.31%, respectively. This may be due to the strong alkalinity of the low modulus, which accelerates the activation reaction of the SMSAB paste precursor and leads to fast-setting and fast-hardening characteristics. However, this hinders the migration of free water molecules within the paste and prematurely terminates depolymerisation and condensation reactions, resulting in a gradual decrease in later strength. It is worth noting that this trend changes when the water glass modulus is 1.2. The 3-day compressive strength of SMSAB paste decreased first and then increased with the increase in alkali content. However, the 7-day and 28-day compressive strength of SMSAB paste increased first and then decreased with the increasing alkali content. This is likely due to Na^+^ being adsorbed on the surface of SiMnS particles and reacting with Si-O or Al-O groups to form relatively low-strength amorphous N-A-S-H gels, resulting in decreased production of C-A-S-H gels and C-S-H gels in the hydration products [36]. Therefore, the mechanical strength of the SMSAB paste decreased.

#### 3.2.2. Flexural Strength

**Figure 6:**
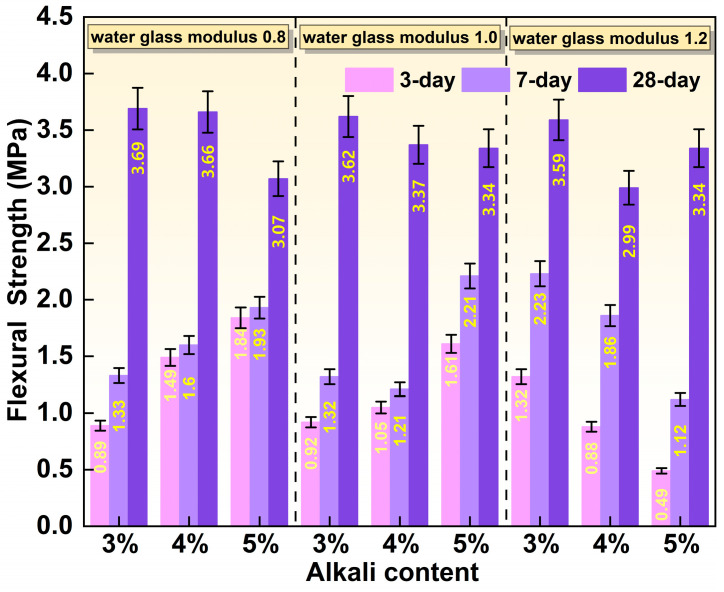
Effect of water glass modulus and alkali content on the flexural strength of SMSAB paste.

Figure 6 displays the effects of water glass modulus and alkali content on the flexural strength of SMSAB paste at different ages. It can be seen that when the modulus of water glass was 0.8 and 1.0, the change in flexural strength of SMSAB paste at 3-day, 7-day and 28-day followed a similar pattern to that of compressive strength. The main reason for this phenomenon can be explained by the compressive strength development law. However, different from the change pattern of compressive strength, when the modulus of water glass was 1.2, the 3-day and 7-day flexural strength of SMSAB paste decreased with increasing alkali content. Specifically, when the alkali content increased from 3% to 5%, the 3-day flexural strength decreased by 33.33% and 62.88%, and the 7-day flexural strength decreased by 16.58% and 49.78%, respectively. This decrease may be related to the increased brittleness of SMSAB paste at a high sodium silicate modulus.

It should be noted that the mechanical results reported here were obtained from paste specimens. Therefore, they should be interpreted as binder-level strength indicators rather than direct pavement-layer performance. Although the optimised SMSAB paste showed relatively high compressive strength, actual road materials contain an aggregate skeleton and are affected by aggregate gradation, binder content, compaction, curing condition, shrinkage, fatigue behaviour and environmental exposure. Thus, the present strength results indicate the reactivity and mechanical potential of the hybrid binder, while the performance of aggregate-containing SMSAB road mixtures still requires further verification.

### 3.3. Microscopic Characterisation

#### 3.3.1. XRD Analysis

**Figure 7:**
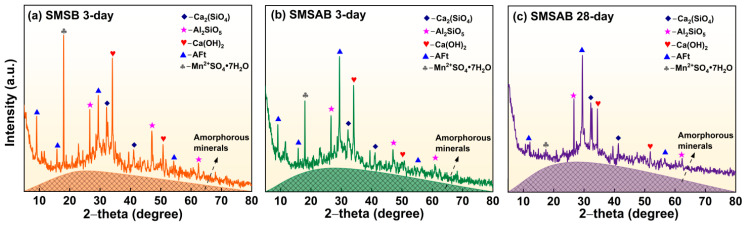
XRD diffractograms of SMSAB paste and SMSB paste.

Figure 7 displays the phase evolution of 3-day and 28-day SMSAB paste with 4% alkali content, and 1.0 water glass modulus, with the XRD pattern of 3-day SMSB paste serving as the control group. It can be seen that the presence of anorthite (Ca_2_SiO_4_), ettringite (AFt), aluminium silicate (Al_2_SiO_5_), Ca(OH)_2_, mallardite (Mn^2+^SO_4_·7H_2_O), and amorphous mineral phases were identified as the dominant mineral phases in SMSB paste and SMSAB paste. There was an obvious “diffuse peak” in the range of 25–38° in all samples, corresponding to the amorphous minerals phase, which is mainly a mixture of C-S-H gels, C-A-S-H gels, and hydrated calcium sulphoaluminate (AFm). Figure 7a presents the peak intensity of Mn^2+^SO_4_·7H_2_O in the 3-day diffractogram of the unactivated SMSB paste was relatively larger, which is due to the presence of a certain amount of silicate and sulphate in the hydration products of SMSB paste, and the heavy-metal ions in SiMnS were formed alkaline salt precipitation under alkaline environment, and Mn^2+^ combines with sulphate to generate a large amount of Mn^2+^SO_4_·7H_2_O, then resulting in a strong diffraction peak [37]. It should be noted that Mn^2+^SO_4_·7H_2_O itself is not toxic, but it contains Mn^2+^ and long-term exposure to sulphate is harmful to the human body. Fortunately, by contrast, the peak intensity of Mn^2+^SO_4_·7H_2_O in the 3-day diffractogram of alkali-activated SMSAB paste was significantly reduced (see Figure 7b). In addition, the area of the amorphous phase increased and the peak value of the Ca(OH)_2_ phase decreased, indicating that alkali activation promoted the dissolution of active substances in the precursor and generated more amorphous gels.

Figure 7c shows that with increasing curing age, the peak strength of Mn^2+^SO_4_·7H_2_O in the 28-day diffraction pattern of SMSAB paste was very small. The decrease in the Mn-bearing crystalline signal may be related to Mn retention within the reaction products or to the reduced crystallinity/content of Mn-bearing phases. Mn^2+^ may participate in charge-balancing interactions with negatively charged [AlO_4_]^−^ units in the gel structure by partially replacing Na^+^ or Ca^2+^, but this interpretation cannot be directly confirmed by XRD alone. Under alkaline conditions, Mn-bearing precipitates may also form and contribute to Mn retention [38]. However, no independent Mn oxide phase was clearly detected by XRD in this study. Therefore, the formation of Mn oxide should be regarded as a possible pathway rather than direct phase evidence. In addition, the area of amorphous phase in the 28-day diffractogram of SMSAB paste was further increased, the diffraction peaks of Al_2_SiO_5_ and AFt phases became widened and enhanced, and the peak of Ca(OH)_2_ phase decreased, indicating that the dissolution efficiency of the vitreous structure in the precursor was further improved, and a large number of C-S-H gels and C-A-S-H gels were generated in the polymerisation reaction. This explains the apparent increase in mechanical strength of SMSAB paste at 28 days.

#### 3.3.2. SEM Analysis

**Figure 8:**
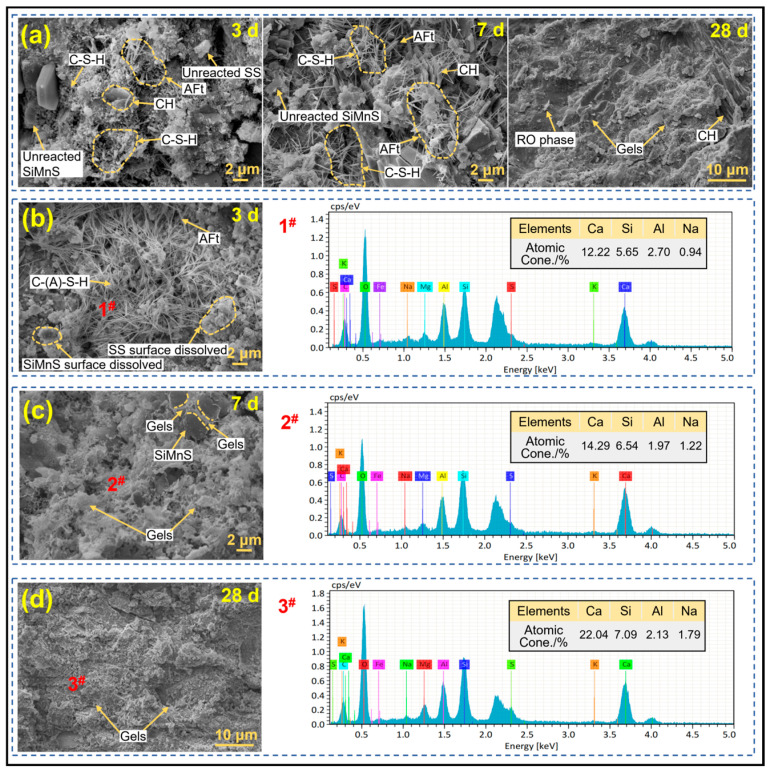
SEM and EDS of SMSB paste and SMSAB paste: (**a**) SMSB, (**b**) 3-day SMSAB, (**c**) 7-day SMSAB, and (**d**) 28-day SMSAB.

Figure 8 displays the microstructures together with elemental compositions of reaction products from the 3-day SMSAB paste with 4% alkali content and 1.0 water glass modulus, alongside the 3-day SMSB paste as the control group. The tables in the energy-dispersive spectra show the atomic percentages at the selected analysis points. As shown in Figure 8a, the 3-day SMSB paste contained fewer flocculent C-S-H gels and rod-like AFt crystals, while more voids were observed. With the increase in curing age, C-S-H gels and rod AFt developed and grew in the 7-day SMSB paste, and they interlaced with each other, leading to a more compact microstructure of the system. The gel products in the 28-day SMSB paste encapsulated the precursor particles to form a dense microstructure. However, for the alkali-activated material system, the microstructure changed significantly. As shown in Figure 8b, it can be observed that a large number of fibrous C-S-H gels and rod-like AFt were interspersed with each other in the 3-day SMSAB paste, filling the gap between the precursor particles that had not yet undergone the alkali activation reaction, and forming a dense spatial network structure in the early stage. In addition, SiMnS and SS particles with signs of surface erosion were also observed. As the curing age increased, the gel network further became denser and more tightly bonded to the voids between SiMnS particles (see Figure 8c). The matrix was completely covered by a large number of gels in the 28-day SMSAB paste, forming a highly dense microstructure (see Figure 8d), which supports the view regarding the high strength of SMSAB paste. The findings indicate that under the condition of water glass as alkali activator, the number of reaction products increased, which improved the macroscopic strength of SMSAB paste.

Figure 8b–d also presents the representative spectrum of EDS point analysis of the main reaction products. According to the EDS results, the elemental composition of the reaction products in the SMSAB paste was mainly O, Ca, Si, Al, Na, etc., which are the main components of the alkali-activated gel backbone [39]. Notably, a small amount of Mn (0.11%) was detected in the reaction products, suggesting that Mn-bearing species were present in the hardened SMSAB matrix. However, EDS cannot determine the oxidation state of Mn or prove its structural incorporation. Therefore, this result is interpreted only as supporting evidence for possible Mn retention within the matrix. The curing time was extended from 3-day to 28-day, the content of Ca in the reaction product increased from 12.22 to 22.04, the Ca/Si ratio increased from 2.13 to 3.11, and the Ca/(Al + Si) ratio increased from 1.46 to 2.39, indicating that the higher degree of reaction in the SMSAB paste may lead to a higher amount of C-(A)-S-H gels and calcium-rich cross-linked gels (C-N-A-S-H). This is because in an alkali-activated environment, the [Al(OH)_4_]^−^ and [Si(OH)]^4−^ monomers in the precursor are released from the aluminosilicate phase. Subsequently, [Si(OH)]^4−^ reacts with Ca(OH)_2_ to generate C-S-H gels, and part of the Al enters the C-S-H gels to generate C-A-S-H gels. However, the replacement of Si for Al generates a negative charge vacancy, which makes the Na^+^ provided by the activator easily adsorbed, resulting in the formation of C-N-A-S-H gels [40,41]. Na/(Al + Si) increased from 0.11 to 0.19, indicating that the content of N-A-S-H gel in SMSAB paste increased. In addition, due to the high content of all the main elements (Na, Ca, Si and Al) involved in the gel composition, the likelihood of a high degree of cross-linked coupling of C-(A)-S-H gels and N-A-S-H gels in the SMSAB paste was further confirmed. Previous studies have reported that the reaction products in the alkali activation system are mainly C-A-S-H gels, N-A-S-H gels and C-N-A-S-H cross-linked gels, accounting for 60% to 66% of the slurry volume [42,43]. Among them, the reaction products are mainly C-A-S-H gels and C-N-A-S-H cross-linked gels, supplemented by N-A-S-H gels [44]. Similar results were observed in the current study.

#### 3.3.3. FTIR Analysis

**Figure 9:**
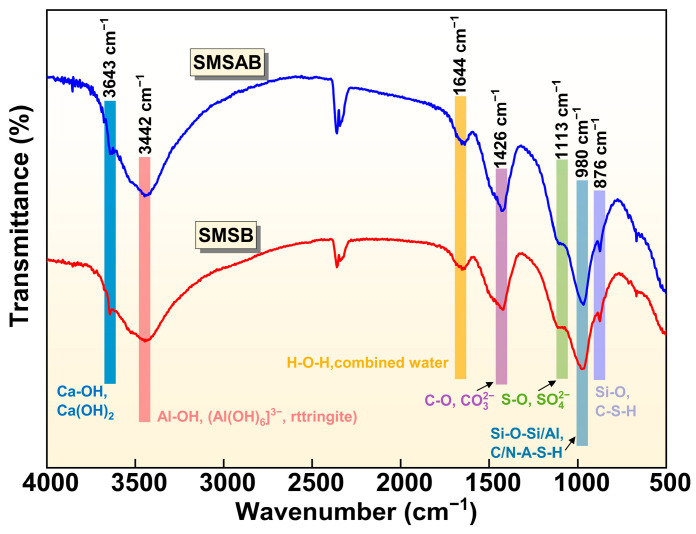
FTIR curves of SMSAB paste and SMSB paste at 3-day ages.

Figure 9 presents the infrared spectrum of 3-day SMSAB paste with 4% alkali content and 1.0 water glass modulus, alongside the infrared spectrum of 3-day SMSB paste as the control group. It can be seen that the infrared spectra of SMSAB paste and SMSB paste at 3 days of hydration were the same, but the intensity and width of the characteristic peaks were slightly different, indicating that the hydration products only changed in content, which was consistent with the XRD analysis results. In Figure 9, the absorption band occurring at 3465 cm^−1^ was associated with the Ca-OH stretching vibration [39], which corresponds to a characteristic band of Ca(OH)_2_, and it was weaker in SMSAB than in SMSB paste, indicating that the alkali activation system consumed Ca^2+^ and polymerised with dissolved vitreous to generate additional gel products. The absorption band at 3442 cm^−1^ corresponds to the Al-OH functional group [45]. The intensity of the characteristic bands belonging to AFt in the SMSAB paste increased and moved to a higher wave number, indicating that the generation and degree of polymerisation of AFt increased, which is consistent with the XRD results. The absorption band at approximately 1644 cm^−1^ corresponds to the bending vibration mode of the H–O–H bond, and the absorption band at 1113 cm^−1^ was the S-O bond in SO_4_^2−^ [24]. The absorption band at 1426 cm^−1^ was caused by the asymmetric stretching vibration of the C-O bond in planar CO_3_^2−^, indicating that CO_2_ was absorbed by the SMSB paste during curing [46]. The strongest band at approximately 980 cm^−1^ corresponded to the asymmetric stretching vibration of Si-O-T (T = Si, Al), which was assigned as a characteristic band of the aluminosilicate gels [47,48]. Generally, the absorption band wavenumber of Si-O-T (T = Si, Al) is closely related to Si/Al in the gel: when Si/Al increases (or decreases), the absorption band shifts to a higher (or lower) wavenumber due to the lower force constant of Al-O [49,50]. For the SMSAB system, the Si-O-T (T = Si or Al) absorption band shifted from 977 to 970 cm^−1^. The shift in the Si-O-T band from 977 to 970 cm^−1^ may indicate a change in the local bonding environment of the aluminosilicate gels. One possible explanation is that Mn species interacted with non-bridging oxygen sites, leading to Mn-related bonding environments such as Si-O-Mn type linkages [51,52]. However, FTIR band shifts alone cannot provide definitive evidence for Si-O-Mn bonding. Therefore, this interpretation should be considered together with the leaching results, SEM-EDS observations and XRD evidence. Another possible explanation is that Mn species may contribute to charge-balancing interactions near [AlO_4_]^−^ units within the SMSAB gel framework. The asymmetric stretching vibration band at 876 cm^−1^ corresponds to the Si-O in C-S-H gels [53]. The intensity of the Si-O absorption band increased in SMSAB paste, indicating that the amount of amorphous gel products increased in SMSAB paste, which was consistent with the increase in the amorphous area observed by XRD. This also explains the observed significant increase in compressive strength.

#### 3.3.4. MIP Analysis

**Figure 10:**
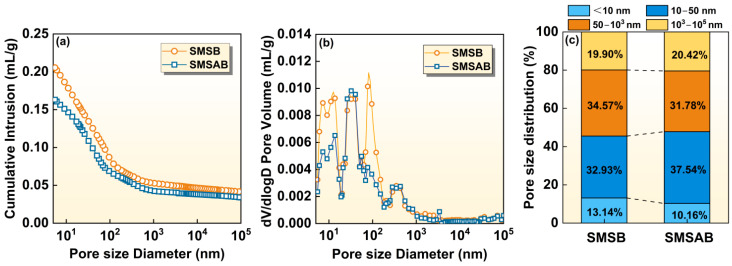
MIP analysis of SMSB and SMSAB pastes: (**a**) pore size cumulative distribution curve, (**b**) pore size differential distribution curve, and (**c**) pore volume percentage of the pore with different pore size diameters.

Figure 10 presents the pore size structure and distribution of the SMSB and SMSAB paste at 3-day age, determined by MIP analysis, and their pore structure characteristics are given in Table 3. From Figure 10a, the relationship between the cumulative intrusion volume and the pore size diameter is plotted. The cumulative intrusion values of SMSB and SMSAB paste were 0.2055 mL/g and 0.1631 mL/g, porosity values were 31.89% and 26.09%, and the total pore area values were 27.96 m^2^/g and 20.23 m^2^/g, respectively (see Table 3). Compared with SMSB paste, the pore structure characteristics of SMSAB paste were lower, which confirms that it exhibited a relatively denser microstructure. The pore structure (see Figure 10b) of the paste showed that the size of most of the pores was below 100 nm. Generally, in cementitious materials, pores are classified into four types [54,55,56,57]: gel pores (pore size < 10 nm), transitional pores (pore size, 10–50 nm), capillary pores (50–10^3^ nm), and coarse pores (pore size, 10^3^–10^5^ nm). Among them, gel pores and transitional pores influence the durability, strength and shrinkage, whereas coarse pores and capillary pores influence the strength and permeability of the matrix. However, the small pore size (<50 nm) is closely related to the reaction products of the paste. The volume of small-sized pores in SMSB paste was 46.07%, and that of SMSAB paste was 47.7%, which was increased by 1.63%, indicating that a higher amount of gel phases was formed in SMSAB paste, contributing to its strength development.

### 3.4. Analysis of Pore Fractal Characteristics

The area, volume and shape of pores have significant fractal characteristics, and the fractal dimension reflects the effectiveness of complex pores in space. In this study, the Menger sponge model, the Neimark model and the model based on the law of thermodynamics were used to determine the analytical dimensions of SMSB paste and SMSAB paste.

Friesen and Mikula [58] proposed a method to calculate the fractal dimension based on the Menger sponge equation to simulate the pore structure of particles and the coexistence of different pore sizes. The pore sizes of different sizes can be completely and continuously characterised. Fractal dimension DK as shown below:

(1) $$\mathrm{lg}(-dV/dr)\propto (2-D_{K})\mathrm{lg}r$$

where *V* is the cumulative volume intruded by mercury, *r* is the pore size, and *k* is the proportional coefficient.

The Neimark model was derived by combining the proposed formula and the law of energy conservation [59]. The Neimark model can calculate the surface area of the pores intruded by mercury:

(2) $$S=1/\gamma \cos\theta \int_{0}^{V_{P}}PdV$$

where *γ* is the surface tension of mercury (0.458 N/m), *θ* is the contact angle between mercury and pore surface in porous medium (130°), *S* is the pore surface area of the pores intruded by mercury, and *V* is the volume of mercury injection.

Then, according to the necessary and sufficient condition Equation (4) and the law capillary Equation (5) [60], the fractal dimension is determined by establishing the relationship between S and P:

(3) $$dV/dr\propto r^{2-D_{N}}$$

(4) P=2γcosθ/r

(5) $$\mathrm{lg}S\propto (D_{N}-2)\mathrm{lg}P$$

where *r* is the pore radius, *P* is the mercury inlet pressure, and *D_N_* is the fractal dimension.

Zhang and Li [61] proposed a thermodynamic analysis model, considering that the work done by the applied pressure on mercury is equal to the increase in the surface energy of mercury:

(6) $$\int_{0}^{V}P\mathrm{dV}=-\int_{0}^{S}\sigma \cos\theta dS$$

The internal pore surface area S of the paste is related to the pore diameter d and the cumulative volume intruded by mercury V, and the discretisation of the fractal dimension can be obtained:

(7) $$\sum_{i=1}^{n}{\bar{P}}_{i}\Delta V_{i}=kr_{i}^{2}{\left(V_{i}^{1/3}/r_{i}\right)}^{D_{H}}$$

where *P_i_* is the pressure of the ith mercury injection, Δ*V_i_* is the amount of the ith mercury injection, *k* is the material parameter, *r_i_* is the pore radius corresponding to the pressure of the ith mercury injection, and Vi is the cumulative amount of the ith mercury injection.

(8) $$W_{i}=\sum_{i=1}^{n}{\bar{P}}_{i}\Delta V_{i}$$

(9) $$Q_{i}=V_{i}^{1/3}/r_{i}$$

Then, substitute Equations (9) and (10) into (8), and take logarithms of both sides of the following equation:

(10) $$\ln(W_{i}/r_{i}^{2})=D_{H}\ln Q_{i}+C$$

where *W_i_* is the cumulative surface energy, *Q_i_* is the function of ri and *V_i_*, and *C* is a constant. The fractal dimension, *D_H_*, can be obtained by the above equation.

**Figure 11:**
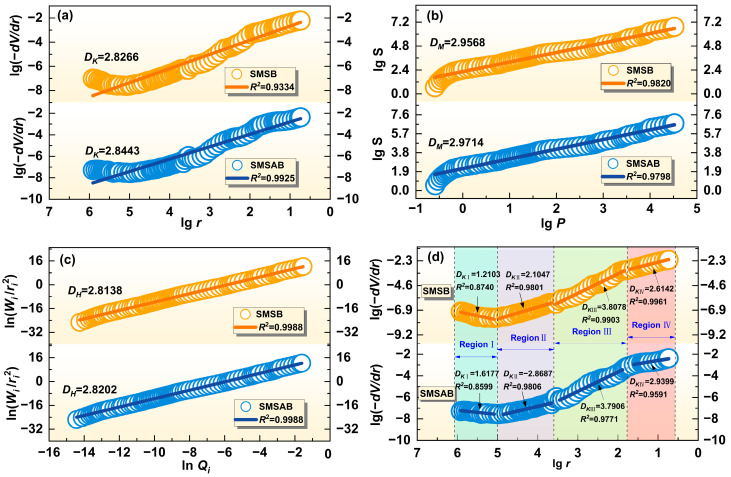
Analysis of pore fractal characteristics: (**a**) Menger sponge model, (**b**) Neimark model, (**c**) thermodynamic model, and (**d**) multifractal characteristics based on the Menger sponge model.

Figure 11 displays the three fractal dimension models of SMSB and SMSAB paste. As shown in Figure 11a–c, the three models have a strong correlation in the whole pore size range, with R2 all greater than 0.9, indicating that the pore structure of SMSB and SMSAB paste has obvious fractal characteristics. The fractal dimension *D*_K_ of the Menger sponge model was between 2.8266 and 2.8443, the fractal dimension *D*_M_ of the Neimark model was between 2.9568 and 2.9714, and the fractal dimension *D*_H_ of the thermodynamic model was between 2.8138 and 2.8202, all of which fall within a reasonable range of 2–3. Based on the fractal theory, the pore structure tends to be smooth when the fractal dimension of the unit is smaller (close to 2). When the fractal dimension is larger (close to 3), the pore space structure is more complex, and the pore space occupying capacity is enhanced. In these three models, the fractal dimension of SMSAB paste was higher than that of SMSB paste, indicating that the pore distribution of SMSAB paste was complex and the reaction products were well developed [62,63]. In other words, the proportion of gelled pores and transitional pores in the pore structure was relatively high. In addition, compared with the Menger sponge model and the Neimark model, the fitting curve of the thermodynamic model was basically unchanged throughout the diameter range. In other words, the in (*Q_i_*)–In (*W_i_*/*r_i_*^2^) coordinate system could be fitted with a straight line, and R2 was greater than 0.99 (see Figure 11c). This means that there was only one fractal dimension in the whole diameter range [64]. Therefore, the thermodynamic model is the most suitable to determine the fractal dimension in an alkali-activated gel system.

To further investigate the multifractal characteristics of the pore structure of SMSB and SMSAB pastes, the Menger sponge model and the Neimark model were used. Taking the Menger sponge model as an example, the fractal characteristics of the paste in four regions (region I (10^3^–10^5^ nm), region II (50–10^3^ nm), region III (10–50 nm), and region IV (<10 nm)) were studied. As shown in Figure 11d, the coarse pores DKI < 2 are in region I, and the transition pores > 3 are in region III. A fractal dimension outside the range of 2–3 is considered invalid. Therefore, regions I and III have no fractal characteristics. In regions II and IV with fractal characteristics, the fractal dimension of gelled pores was higher than that of capillary pores, because the gelled pores include the intergranular pores, micropores and interlayer pores of the reaction products, resulting in a more complex gelled pore structure. In addition, compared with the SMSB paste, the DKIV (2.9399) of the SMSAB paste was larger, indicating that additional gel products were formed inside the structure under alkali activation conditions. This was consistent with the results of XRD analysis.

### 3.5. Heavy-Metal Leaching Risk

Hybrid binders prepared with industrial solid wastes may develop dense microstructures that help reduce heavy-metal leaching. However, due to the high Mn content in SiMnS, previous studies have shown that using SiMnS as an auxiliary cementitious material in acidic environments may lead to excessive Mn ion leaching [65]. In this study, two heavy-metal leaching test methods, HVM and SNM, were used to evaluate the SMSB and SMSAB pastes. HVM was used to simulate the leaching behaviour of solid waste under surface water or groundwater conditions, while SNM was used to investigate the leaching characteristics of solid waste and its derived materials under acidic conditions.

**Figure 12:**
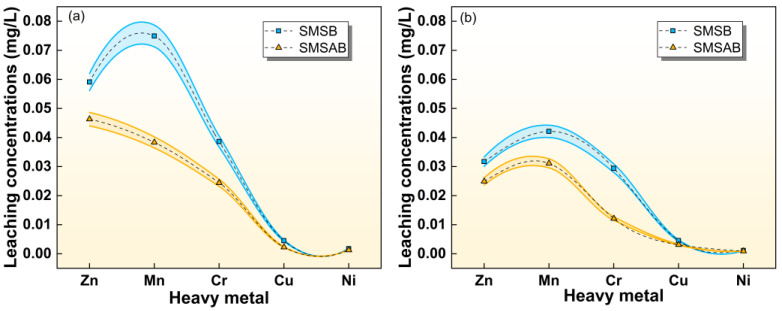
Heavy-metal leaching concentrations of SMSB and SMSAB paste: (**a**) HVM experiments, (**b**) SNM experiments.

Figure 12 displays the heavy-metal leaching behaviour of the two paste samples. The results show that the leaching concentrations of Zn, Mn, Cr, Cu, and Ni in both paste samples comply with the Grade III groundwater standard [66], i.e., Zn < 1.00 mg/L, Mn < 0.10 mg/L, Cu < 1.00 mg/L, and Ni < 0.02 mg/L. The heavy-metal leaching in the SNM test was lower than that in the HVM test. On one hand, the use of larger particle size samples in the SNM test reduces the specific surface area, thereby decreasing the contact with the acidic solution, which in turn reduces heavy-metal leaching and slows down the migration rate of internal ions [67,68]. On the other hand, under acidic conditions, H+ ions in the solution may react with the solid-phase materials, forming a dense passivation layer that effectively inhibits further leaching of heavy metals [69]. Moreover, the heavy-metal leaching in the SMSAB paste was significantly lower than in the SMSB paste, especially for Mn. In the HVM and SNM tests, the Mn leaching in SMSAB paste decreased by 48.87% and 26.13%, respectively.

The reduction in Mn leaching from SMSAB may be related to both physical and chemical retention pathways. From the physical perspective, the dense gel matrix and refined pore structure may hinder the outward diffusion of Mn-bearing species [64]. From the chemical perspective, Mn species may interact with the aluminosilicate or calcium aluminosilicate gel structure through charge-balancing interactions, especially near negatively charged [AlO_4_]^−^ units. This interpretation is consistent with the reduced Mn leaching and the presence of Mn in the reaction products detected by SEM-EDS, but it cannot be regarded as direct proof of lattice incorporation or Mn speciation. In addition, Mn-bearing crystalline phases or precipitates may contribute to Mn retention under alkaline conditions. XRD detected mallardite in the paste, while no independent Mn oxide phase was clearly identified. Therefore, Mn oxide formation should be treated as a possible pathway rather than direct evidence. FTIR band shifts were consistent with changes in the gel bonding environment and possible Mn-related interactions, but they cannot independently prove Si-O-Mn bonding [70].

**Figure 13:**
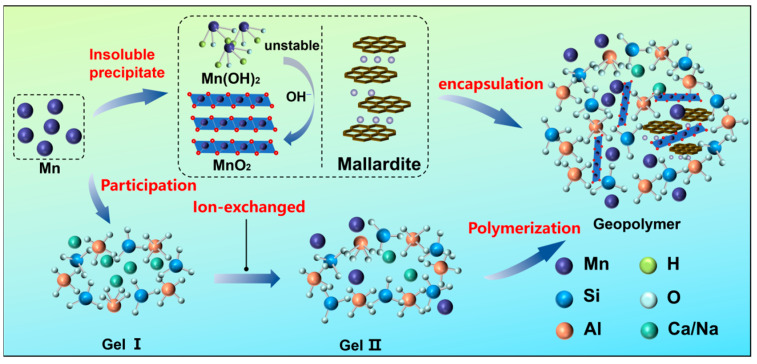
Proposed Mn leaching-reduction pathways in SMSAB paste.

In conclusion, the present leaching results demonstrate that SMSAB reduced Mn leaching under the adopted HVM and SNM test conditions. Based on the combined evidence from XRD, SEM-EDS, FTIR and MIP, the reduction in Mn leaching may be associated with physical encapsulation by the dense gel matrix, charge-balancing interactions within the gel structure and the presence of Mn-bearing phases such as mallardite. However, the current evidence does not definitively determine the oxidation state, chemical speciation or exact binding environment of Mn. In particular, EDS cannot determine Mn valence, FTIR band shifts alone cannot prove Si-O-Mn bonding, and Mn oxide formation was not directly confirmed by XRD. Therefore, Figure 13 presents proposed Mn leaching-reduction pathways rather than a fully proven immobilisation mechanism. Direct Mn speciation analysis, such as XPS, XANES or sequential extraction, together with long-term leaching tests, is still needed to verify the chemical state and long-term stability of Mn in SMSAB.

### 3.6. Simplified Carbon-Footprint Assessment

Reducing CO_2_ emissions is one of the motivations for developing the proposed hybrid SiMnS–SS–FA–OPC-activated binder. In this study, the environmental performance of SMSAB was evaluated using a simplified cradle-to-gate material carbon-footprint calculation rather than a full life-cycle assessment. To enable comparison with published mortar-based reference systems, a mortar-scale carbon-emission scenario was calculated using the optimised SMSAB binder and fine aggregate, as shown in Table 4. The functional unit was defined as 1 m^3^ of SMSAB mortar-scale mixture. The system boundary included the embodied CO_2_ emissions associated with the raw materials, including OPC, SiMnS-SS, FA, NaOH, Na_2_SiO_3_, water and fine aggregates. Transportation, laboratory grinding energy, mixing and curing energy, construction processes, service life, maintenance and end-of-life stages were not included. Therefore, the results should be interpreted as a material-level comparative carbon indicator rather than a complete LCA result. It should also be noted that the mechanical strength reported in this study was measured on paste specimens, while the carbon-emission comparison was normalised at the mortar-scale system level for eco-efficiency discussion.

As shown in Table 4, the total material-related CO_2_ emission of SMSAB was 258.833 kg CO_2_-eq per m^3^. OPC was the dominant contributor, producing 184.8 kg CO_2_-eq and accounting for approximately 71.4% of the total emission. In addition to OPC, alkaline activators, including NaOH and Na_2_SiO_3_, were also important contributors to carbon emissions. NaOH and Na_2_SiO_3_ together accounted for approximately 14.21% of the total carbon emission, which can be attributed to the high energy consumption required in their production processes [70]. However, reducing the alkali content only for the purpose of lowering CO_2_ emissions may lead to a decrease in the strength development of alkali-activated systems [71]. In contrast, SiMnS, SS and FA are industrial by-products that do not require additional high-temperature processing such as calcination within the adopted system boundary, and their combined carbon contribution accounted for approximately 7.62% of the total emission. Therefore, the carbon reduction in SMSAB depends not only on replacing part of OPC with industrial by-products, but also on balancing activator dosage, mechanical performance and environmental benefit.

**Table 4:** Details of carbon emissions per 1 m^3^ of SMSAB mortar produced.

Mixture Type	Materials	Mass of 1 m^3^ Mixture (t)	Mass of Each Material in 1 m^3^ Mixture (t)	Carbon Emission Factor of Each Material Extraction (kgCO_2_-eq/t)	CO_2_ Emissions from Each Material (kg)	Total CO_2_ Emissions (kg)	Carbon Emission Factor Source
SMSAB	OPC	1.8384	0.231	800	184.8	258.833	[72]
	SiMnS-SS		0.126	143	18.018		[73]
	FA		0.063	27.1	1.7073		[74]
	NaOH		0.005	1601	8.005		[75]
	Na_2_SiO_3_		0.019	1514	28.766		[72]
	Water		0.1344	0.168	0.023		[73]
	Fine Aggregates		1.26	13.9	17.514		[72]

To comprehensively evaluate the strength-normalised carbon performance of the optimised SMSAB system, this study used carbon intensity as an evaluation indicator, with the calculation formula shown in Equation (11). Carbon intensity measures the carbon emissions per unit strength, serving as an important metric for assessing the environmental performance of construction materials. The carbon emission and carbon intensity of the optimised SMSAB system were compared with OPC-40, OPC-60 and OPC-100 mortars reported by Liao et al. [76] and Bahri et al. [77], as well as WLSP-30 reported by Ibrahim et al. [78].

Carbon intensity = carbon emission/*f_c_* (11)

where carbon intensity is the coefficient for carbon emission evaluation of mortar (kg·(m^3^·MPa)^−1^), and fc is the mortar compressive strength.

**Figure 14:**
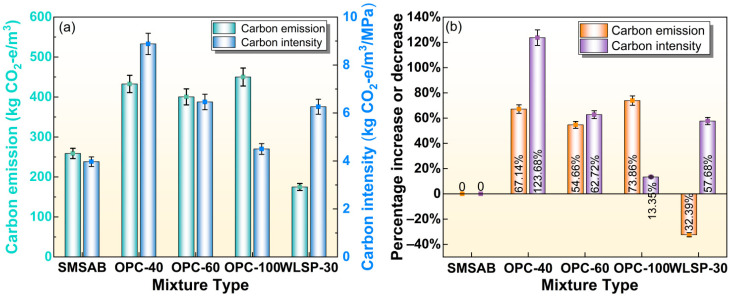
Carbon footprint: (**a**) carbon emissions and carbon intensities, (**b**) percentage increase or decrease in carbon emissions and carbon intensities of the mortar. Note: OPC-X sample indicates that the strength grade of OPC-based mortar is X MPa, and WLSP-30 sample is alkali-activated limestone powder mortar containing 30% OPC.

Figure 14 presents the comparison of carbon emissions and carbon intensity between SMSAB and the reference mortar systems. Compared with traditional cement-based reference mortars, namely OPC-40, OPC-60 and OPC-100, the carbon emissions of SMSAB were reduced by 67.13%, 54.66% and 62.72%, respectively, indicating material-level carbon reduction potential. This reduction was mainly attributed to the partial replacement of OPC by lower-carbon industrial by-products, including SiMnS, SS and FA, and to the different material route of the hybrid activated system compared with conventional cement-based materials [79]. Compared with WLSP-30, the total carbon emission of SMSAB increased by 32.39%. Nevertheless, the carbon intensity of SMSAB was reduced by 57.68%, indicating that SMSAB achieved a relatively favourable balance between mechanical strength and material-related CO_2_ emission. This strength-normalised carbon performance can be attributed to the optimised raw material composition and alkali activator usage, which resulted in relatively high mechanical performance while maintaining a lower material carbon burden [78,80].

However, this comparison has clear limitations. The reference systems OPC-40, OPC-60, OPC-100 and WLSP-30 were taken from published mortar studies, whereas the mechanical performance of SMSAB in this work was obtained from paste specimens. These systems differ in aggregate content, binder chemistry, curing regime, specimen geometry and potentially fresh and durability properties. Therefore, the carbon intensity comparison should be interpreted as a strength-normalised eco-efficiency indicator rather than a direct equivalence of physical and mechanical performance. A rigorous comparison for road engineering would require SMSAB mortar or concrete mixtures with comparable aggregate skeletons, curing conditions, workability, shrinkage, elastic modulus, fatigue behaviour and durability under wet–dry or freeze–thaw exposure. Overall, the present simplified carbon-footprint assessment suggests that SMSAB has promising material-level carbon reduction potential, but its full environmental benefit should be further verified under practical mixture design and service conditions.

## 4. Conclusions

This study evaluated an OPC-rich hybrid SiMnS–SS–FA–OPC-activated composite binder, referred to as SMSAB, with emphasis on paste-level mechanical performance, reaction products, pore structure, heavy-metal leaching behaviour and simplified material carbon footprint. The SMSAB paste achieved its best mechanical performance at an alkali dosage of 4% and a sodium silicate modulus of 1.0; after 28 days of curing, the compressive and flexural strengths reached 65.13 MPa and 3.37 MPa, respectively. XRD, SEM-EDS, FTIR and MIP results indicated that the main reaction products included C-(A)-S-H, N-A-S-H and C-N-A-S-H type gels, which contributed to matrix densification and pore refinement. Mn-bearing phases such as mallardite were detected, and these phases may contribute to pore filling and pore refinement. The thermodynamic fractal model provided a suitable description of the pore system, with a fractal dimension *D_H_* of 2.8202 and an *R*^2^ value close to 1. SMSAB showed reduced Mn leaching under the adopted HVM and SNM conditions. Based on the present leaching and microstructural evidence, this reduction may be associated with physical encapsulation, charge-balancing interactions within the gel structure and the presence of Mn-bearing phases. However, direct Mn speciation and long-term leaching stability were not determined in this study. Within the adopted simplified material-level carbon-footprint boundary, SMSAB showed a carbon intensity of 3.97 kg·(m^3^·MPa)^−1^. Overall, the proposed hybrid binder provides a feasible paste-level route for utilising SiMnS and SS while reducing Mn leaching and showing preliminary material-level carbon reduction potential. Nevertheless, the present study remains a paste-level evaluation. Quantitative fresh-state properties, aggregate-level mixture design, shrinkage, long-term durability, wet–dry resistance, freeze–thaw resistance, fatigue performance, environmental exposure resistance, cost, scalability and field performance should be systematically investigated before practical road application.

## Figures and Tables

**Table 1:** Main chemical composition of raw materials (%).

Raw Materials	Mass Fraction (%)									
	SiO_2_	Al_2_O_3_	Fe_2_O_3_	CaO	MgO	K_2_O	Na_2_O	TiO_2_	SO_3_	P_2_O_5_
SiMnS	31.51	21.28	0.82	18.25	5.86	1.72	0.68	10.92	—	2.71
SS	15.26	3.12	20.98	43.69	4.36	0.02	0.13	2.74	1.85	0.39
OPC	16.41	4.14	4.09	65.32	1.48	0.13	0.28	—	—	2.20
FA	47.04	31.42	6.45	4.94	1.23	2.30	0.78	—	1.72	0.78

**Table 2:** The SMSAB paste mixture proportion.

No.	SiMnS (%)	SS (%)	FA (%)	OPC (%)	Water Glass Modulus	Alkali Content (%)	W/S
1	15	15	15	55	0.8	3	0.32
2	15	15	15	55	0.8	4	0.32
3	15	15	15	55	0.8	5	0.32
4	15	15	15	55	1.0	3	0.32
5	15	15	15	55	1.0	4	0.32
6	15	15	15	55	1.0	5	0.32
7	15	15	15	55	1.2	3	0.32
8	15	15	15	55	1.2	4	0.32
9	15	15	15	55	1.2	5	0.32

**Table 3:** Pore structure characteristics of SMSB and SMSAB pastes determined by MIP.

Sample	Total Intrusion Volume (mL/g)	Total Pore Area (m^2^/g)	Porosity (%)	Average Pore Size (nm)	Bulk Density g/mL	Skeletal Density g/mL
SMSB	0.2055	27.96	31.89	32.26	1.5520	2.279
SMSAB	0.1631	20.23	26.09	29.40	1.5994	2.164

## Data Availability

The original contributions presented in this study are included in the article. Further inquiries can be directed to the corresponding author.
